# BUILDING AN INTERDISCIPLINARY CAREER TO ADVANCE HEALTH PROMOTION FOR HEALTHY AGING

**DOI:** 10.1093/geroni/igae098.1022

**Published:** 2024-12-31

**Authors:** Jaime Hughes

**Affiliations:** Wake Forest University School of Medicine, Winston-Salem, North Carolina, United States

## Abstract

As we all continue to age, the availability of evidence-based health promotion programs will be critical to healthy aging. Intervention science and implementation science are two disciplines at the heart of developing and advancing innovative, effective health promotion programs for use within diverse settings, practices, and populations. Both disciplines are highly interdisciplinary, influenced by principles in clinical geriatrics, behavioral medicine, developmental psychology, and organizational psychology. Specific health promotion programs like those focused on the adoption of healthy sleep and activity behaviors may be further influenced by additional principles, including those from behavioral sleep medicine, exercise physiology, and rehabilitation medicine. Cultivating a professional career designing and translating evidence-based health promotion programs such as these requires commitment, collaboration, and creativity. This presentation will feature completed and ongoing work belonging to a diverse research portfolio made possible by interdisciplinary training activities and research support (e.g., NIA, NHLBI, NCCIH), experiences in aging-focused leadership and professional networking (e.g., ESPO/GSA), and more than 15 years of work in applied care settings (e.g., VAHCS, academic learning health systems). This presentation will close with tips on how to access existing NIA and GSA/ESPO resources and supports for cultivating a successful career, including: leveraging professional networks and training opportunities within NIA and across NIH Institutes (ICs), engaging in professional service and leadership, and conducting embedded research across diverse academic, government, and community settings.

